# MicroRNA Expression Profile Identifies High Grade, Non-Muscle-Invasive Bladder Tumors at Elevated Risk to Progress to an Invasive Phenotype

**DOI:** 10.3390/genes8020077

**Published:** 2017-02-17

**Authors:** Sara M. Lenherr, Sheaumei Tsai, Brasil Silva Neto, Travis B. Sullivan, Cara B. Cimmino, Tanya Logvinenko, Jason Gee, Wei Huang, John A. Libertino, Ian C. Summerhayes, Kimberly M. Rieger-Christ

**Affiliations:** 1 Department of Urology, Lahey Hospital and Medical Center, Burlington, MA 01805, USA; sara.lenherr@hsc.utah.edu (S.M.L.); stsai@winhosp.org (S.T.); bneto@hcpa.edu.br (B.S.N); ccimmin@emory.edu (C.B.C); Jason.R.Gee@lahey.org (J.G.); John.A.Libertino@lahey.org (J.A.L.); Ian.C.Summerhayes@lahey.org (I.C.S.); 2 Department of Urology, Hospital de Clinicas de Porto Alegre, School of Medicine, Universidade Federal do Rio Grande do Sul, Porto Alegre 90035-003, Brazil; 3 Cell and Molecular Biology Laboratory, Lahey Hospital and Medical Center, Burlington, MA 01805, USA; Travis.B.Sullivan@lahey.org; 4 Biostatistics Research, Institute for Clinical Research Health Policy Studies, Tufts Medical Center, Boston, MA 02111, USA; Tanya.Logvinenko@childrens.harvard.edu; 5 Department of Pathology, University of Wisconsin, Madison, WI 53726, USA; WHuang@uwhealth.org; 6 Deceased

**Keywords:** microRNA, non-muscle invasive bladder cancer, progression, intravesical therapy

## Abstract

The objective of this study was to identify a panel of microRNAs (miRNAs) differentially expressed in high-grade non-muscle invasive (NMI; TaG3–T1G3) urothelial carcinoma that progress to muscle-invasive disease compared to those that remain non-muscle invasive, whether recurrence happens or not. Eighty-nine high-grade NMI urothelial carcinoma lesions were identified and total RNA was extracted from paraffin-embedded tissue. Patients were categorized as either having a non-muscle invasive lesion with no evidence of progression over a 3-year period or as having a similar lesion showing progression to muscle invasion over the same period. In addition, comparison of miRNA expression levels between patients with and without prior intravesical therapy was performed. Total RNA was pooled for microarray analysis in each group (non-progressors and progressors), and qRT-PCR of individual samples validated differential expression between non-progressive and progressive lesions. MiR-32-5p, -224-5p, and -412-3p were associated with cancer-specific survival. Downregulation of miR-203a-3p and miR-205-5p were significantly linked to progression in non-muscle invasive bladder tumors. These miRNAs include those implicated in epithelial mesenchymal transition, previously identified as members of a panel characterizing transition from the non-invasive to invasive phenotype in bladder tumors. Furthermore, we were able to identify specific miRNAs that are linked to postoperative outcome in patients with high grade NMI urothelial carcinoma of the bladder (UCB) that progressed to muscle-invasive (MI) disease.

## 1. Introduction

Urothelial carcinoma of the bladder (UCB) is the fifth most commonly diagnosed cancer in the United States, with a predicted incidence of 76,960 new cases and an estimated mortality of 16,390 individuals in 2016 [1]. Transitional cell carcinoma (TCC) is the most common form of UCB and can be divided into two groups defined by distinct behaviors and different molecular profiles [2,3]. The first group encompasses low-grade tumors which are always papillary and usually non-invasive (Ta) while the second group consists of high-grade tumors, which can be either papillary or non-papillary and are often invasive, either into the lamina propria (T1) or muscularis propria (T2). Non-muscle invasive (NMI) tumors account for 75%–80% of bladder neoplasms, while the remaining 20%–25% are already invasive or metastatic at the time of presentation [3]. 

NMI (Ta, T1 and in situ) tumors can be effectively treated via transurethral resection and intravesical agents, although recurrence is common [2]. Between 50% and 70% of patients affected with NMI tumors will have one or more recurrences after initial treatment, and about 10%–30% of those patients will progress, presenting at a later date with a more aggressive lesion [3]. Multiple recurrences, multiple lesions (>3), size of the tumor (>3 cm), T1 or high grade disease and the presence of in situ carcinoma are risk factors for either recurrence or progression [3,4]. Therefore, patients at risk for recurrence and/or progression undergo regular surveillance by cystoscopy and urine cytology. A systematic review analyzing a total of 19 trials and over 3000 high-risk NMI patients showed that events related to progression occur early and survival is poor in this specific group [5]. Given that 50% of bladder cancers on average are fatal once they become muscle-invasive [2], the recent American Urologic Association guidelines recommend radical cystectomy to be performed for high-risk NMI disease, looking for a survival benefit, despite the morbidity associated with the surgery [6]. 

The goal of bladder cancer screening is to improve outcomes by facilitating the detection and treatment of early-stage tumors which are destined to become muscle-invasive cancers. At this moment, there is no clinical or laboratory marker that can definitively predict which patients will eventually progress to muscle-invasive (MI) disease. A molecular marker with this ability could indicate the need for radical treatment such as cystectomy to be performed months or even years before progression providing an enormous survival benefit. On the other hand, patients with high-grade disease and no risk of progression would be spared of the morbidity and mortality of the surgery as well as preservation of their native bladders. 

In addition, there has been consideration of the reclassification of T1 lesions with respect to the depth of invasion characterized as in the subepithelial connective tissue or adjacent to the muscle, once again indicative of different invasive potential [7,8]. Grade has also been shown to impact progression-free survival [9]. Although early cystectomy is an option in patients with high grade Ta or T1 bladder tumors the standard treatment of these tumors is transurethral resection (TURBT) followed by intravesical therapy, typically with Bacille-Calmette-Guerin (BCG). The patient is then followed through long-term cystoscopic surveillance with a repeat biopsy where disease progression can be assessed [6]. It is reported that up to 25% of patients with high-grade Ta lesions will progress to muscle-invasive disease [10,11], and under-staging of T1 lesions is reported to occur in ~20% of patients who undergo TURBT restaging [12,13]. It is clear that these patients pose a significant clinical challenge for the practicing urologist and that there is a need for new biomarkers that can more accurately predict tumor behavior.

There have been numerous reports of genome-wide gene expression profiling of different bladder tumor groups highlighting the expression of clusters of genes indicative of specific tumor type [14,15,16,17,18,19,20,21], tumor behavior [22,23,24,25] or response to chemotherapeutic agents [26,27]. A similar approach has been taken with microRNA (miRNA) profiling where altered expression of specific miRNAs has been linked to bladder tumorigenesis [14,28,29,30,31,32] including predictors of outcome [33,34,35,36] and progression to muscle-invasive (MI) disease [37]. Our group has previously reported a miRNA expression ratio defining the invasive phenotype in bladder tumors [38] and identified an extended panel of miRNAs involved in the transition from a non-invasive to an invasive bladder lesion [39] where many of the miRNAs identified in this new panel have been shown to be involved in the epithelial-mesenchymal transition (EMT), a complex process involved in cancer progression [40,41,42,43,44]. In this study, we sought to identify a miRNA panel that can prognosticate the invasive potential of high grade Ta and T1 lesions, help better understand the mechanisms of this disease, and ultimately serve to guide the clinician in deciding upon the appropriate path for patient management. In addition, miRNA expression was examined in a cohort of patients with prior intravesical therapy in an effort to better understand the molecular mechanisms involved.

## 2. Materials and Methods 

### 2.1. Patient Tissue Samples

We have collected tissue samples under IRB-approved protocols from Lahey Hospital and Medical Center or University of Wisconsin patients diagnosed with urothelial carcinoma of the bladder between 1989 and 2008. Criteria for inclusion were a histologic diagnosis of TaG3 or T1G3 with sufficient tissue material for analysis. The T1G3 tumors were re-resected (re-TURBT) as standard-of-care for accuracy/restaging to confirm absence of muscle invasion, as clinically indicated. Formalin-fixed, paraffin-embedded tumor tissue from patients was retrieved from the archives of the Pathology Department. Patient charts were reviewed for relevant clinical data, including prior history of intravesical therapy, concomitant histologic features and determination of final clinical stage at the most recent follow-up. Exclusion criteria included previous tumors of the upper urinary tract, prior intravesical therapy or systemic chemotherapy, metasynchronous lesions (e.g., carcinoma in situ (CIS) or squamous or adenocarcinoma), previous radiation to the pelvis (including brachytherapy) or less than three years of follow-up for non-progressive tumors. These criteria were placed on samples submitted for microarray analysis and validation by qRT-PCR. Patients with CIS (previous or concomitant) or previous intravesical therapy were included in an expanded sample set to further elucidate differential miRNA expression in progressors vs. non-progressors, using qRT-PCR analysis. Progression was defined as muscle invasion (stage T2 or higher) or metastatic disease and was determined by review of the clinical records. Tumor stage was determined using tumor metastasis nodes (TMN) classification and graded according to the WHO guidelines. Five micron sections from tissue samples were cut and stained with hematoxylin and eosin for the assessment of tumor content. Only samples with >60% tumor content were included in this study.

### 2.2. RNA Extraction

Total RNA was extracted from sequential formalin fixed paraffin embedded (FFPE) sections using the RecoverAll^TM^ Total Nucleic Acid Isolation Kit (Ambion, Houston, TX, USA) according to manufacturer’s protocol. Total RNA was quantified using Quant-IT RNA Assay Kit (Invitrogen, Carlsbad, CA, USA) detected with the Qubit-fluorometer (Invitrogen).

### 2.3. Microarray Analysis

Total RNA from patients was pooled into two groups: (1) TaG3 or T1G3 without clinical progression and (2) TaG3 or T1G3 with progression to ≥T2 disease. An equal quantity of total RNA was used for each pool (11 µg), and each member within a pool contributed an equal quantity of RNA. The RNA was analyzed using miRCURY™ LNA Array microRNA Profiling. Briefly, after quality control was performed to determine RNA integrity, concentration, and content of small RNA, RNA was labeled with Hy3™ and Hy5™ fluorophores using the miRCURY™ LNA Array Power labeling kit (Exiqon, Vedbaek, Denmark) and hybridized to the miRCURY™ LNA Array (Exiqon). Dye swap was performed to ensure data was not dependent on labeling technique. 

Data was obtained as a median of replicated fluorescent signal measurements of the same miRNA from each slide for 1266 miRNAs. Slides were analyzed using Exiqon’s protocol. In addition to the raw data for each data point, an expression matrix was performed containing the normalized log2 transformed Hy3/Hy5 ratios. Normalization included background subtractions with a global Lowess (LOcally WEighted Scatterpoint Smoothing) regression algorithm. Spots were flagged and analyzed for signal quality (empty spot, spot signal less than background, less than optimal spot morphology or saturated spot). Flagged miRNAs were excluded from Exiqon’s analysis. Dye-swap was performed to mitigate labeling bias. 

### 2.4. Quantitative Real Time RT-PCR

qRT-PCR quantification of mature miRNAs was conducted using TaqMan miRNA quantification kits following the manufacturer’s protocol (Applied Biosystems, Foster City, CA, USA). Briefly, 5 ng of total RNA was used for reverse transcription and 0.33 ng of cDNA was used per reaction in real-time PCR analysis. Each qRT-PCR reaction was performed in triplicate. MiRNAs for this analysis were based on the differential expression found in the microarray and previous work by our group predictive of urothelial cancer invasiveness [39]. Normalization of miRNA expression was performed using miR-222-3p as previously described [39], and relative expression levels were calculated using the 2^−∆Ct^ method.

### 2.5. Statistical Analysis

To determine whether the miRNAs reported in this study were predictive of progression to invasive disease we used logistic regression with the progressive status being a response and the log-transformed miRNA measurement as a predictor. For univariate overall and cancer-specific survival analysis we used the product-limit procedure (Kaplan-Meier method), with the surgery as the entry date and the continuous miRNA variables were dichotomized (e.g., low vs. high expression) based on the median level of expression. The log-rank (Cox-Mantel) test was used to compare the survival curves for each variable.

To determine the prognostic value of each miRNA in terms of disease-free and overall survival among Ta/T1G3 bladder cancer patients, we used Cox proportional hazard regression to calculate the hazard ratios (HR) for each model. Other prognostic factors, such as age, therapy, and CIS status were tested in univariate proportional hazards Cox models. However, no significant effect on the hazard of cancer progression was observed; therefore, they were not included in the final models. 

Comparison of miRNA expression levels between all patients (progressors plus non-progressors) with and without prior intravesical therapy as well as comparison of miRNA expression levels and survival status were performed using a t-test or ANOVA with Games-Howell post-hoc analysis, as appropriate. Normality assumption was tested using the Shapiro-Wilks test; in those cases where normality assumption was violated, Wilcoxon-Mann-Whitney non-parametric test was used. A *p*-value < 0.050 was considered significant. 

## 3. Results

### 3.1. Discovery Sample Set

Microarray analysis and initial qRT-PCR validation was performed with two groups of samples, a total of 17 patients, including both non-progressors and progressors. The non-progressors (10 patients) presented with Ta/T1G3 tumors and displayed no evidence of progression to muscle-invasive disease in a three-year follow-up period. The progressors (7 patients) presented with Ta/T1 tumors and displayed progression to muscle-invasive cancer within three years. Table 1A details the two groups and clinical profile of these patients. There was a higher percentage of males in the progressor group. For microarray analysis RNA was pooled for each group, and validation of differentially expressed miRNAs, via qRT-PCR, was performed on individual samples. None of the 17 patients were treated with prior intravesical therapy nor was there evidence that they had CIS prior to, or concurrently, with the tumor analyzed in this study.

### 3.2. Microarray Results for miRNAs Differentially Expressed in Non-Progressive Versus Progressive High-Grade Ta and T1 Lesions: Discovery Sample Set

To study changes in expression levels of miRNAs that might be relevant to progression of UCB, we performed miRNA microarray analysis on high-grade, NMI UCBs that did not progress over a three-year follow up period (Ta–T1) and those that progressed from the NMI state to muscle-invasive (≥T2). Analysis of microarray data from both non-progressive and progressive TaG3 and T1G3 lesions was performed by identification of miRNA showing >1.5 or <−1.5-fold change in expression between groups. Using this criterion, we identified 19 miRNAs displaying differential expression between non-progressive and progressive tumor groups (Table 2).

### 3.3. Validation of Differential Expression of miRNAs Using qRT-PCR: Discovery Sample Set

Validation of differential expression was performed using qRT-PCR of individual samples, initially on the 17 samples used in microarray analysis. Differentially expressed miRNAs were chosen for further analysis based on the microarray results and comprised 5 of the upregulated and 14 of the downregulated miRNAs identified in the microarray. For each of the nineteen miRNAs analyzed the qRT-PCR levels obtained were of a similar level of magnitude between the groups corresponding to the results obtained in microarray analysis (Table 2). The differences observed were statistically significant for five out of the nineteen miRNAs where miR-145-5p was the only significantly up-regulated miRNA while miR-141-3p, -200a-3p, -203a-3p, and miR-205-5p were significantly down-regulated in the progressor group. 

### 3.4. miRNA Expression of an Expanded Sample Set Including Patients with CIS and/or Therapy

**Figure 1:**
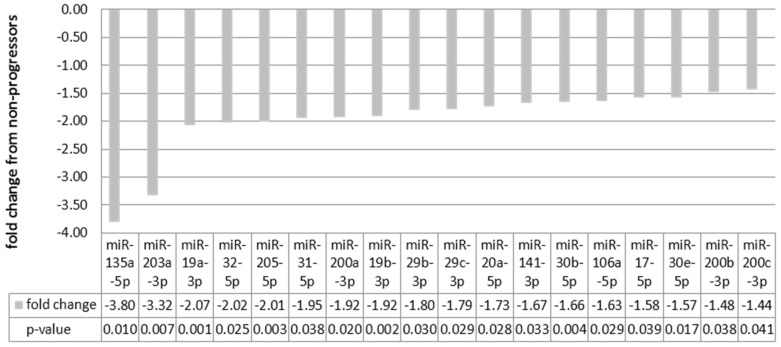
Relative expression within the expanded sample set: qRT-PCR results of significantly differentially expressed miRNAs. Negative fold change values indicate expression was higher in non-progressors.

In order to investigate miRNA expression using a larger sample size and more clinically complex samples, qRT-PCR was performed on an expanded group of 89 Ta–T1/G3 human tumor samples (Table 1B) consisting of 67 non-progressors and 22 progressors inclusive of the 17 samples from the discovery set. Compared to the discovery set, the percentage of males was better balanced between the non-progressor and progressor goups in this expanded set. We also expanded the total number of miRNAs analyzed to 35 (Supplemental Materials Table S1) based on the differential expression found in the microarray and previous work by our group. The original samples used in miRNA microarrays were selected to be as homogeneous as we could obtain i.e., presenting with Ta–T1/G3 lesions alone, while the additional samples analyzed were chosen to have confounding factors that mirror the clinical presentations seen in the majority of patients presenting with high grade Ta/T1 disease. These additional factors included prior therapy and/or CIS. Analysis of the qRT-PCR results revealed that one of the progressor patients was an outlier as their expression levels were, on average, 15-fold higher compared to the other samples in the progressor group. Removal of this sample from the analysis revealed a statistically significant difference between non-progressive and progressive samples for 18 miRNAs (Figure 1). 

Although the additional patients helped to increase the power of the study, they also had confounding factors such as prior intravesical treatment; hence we considered the effect of therapy on miRNA expression. Comparison of miRNA expression levels between patients with and without prior intravesical therapy showed five of the miRNAs tested were significantly different between the two groups. When this analysis was restricted to the progressor-only group, six miRNAs were significant. When restricted to the non-progressor group, there were 19 miRNAs displaying significant expression profile change in the different therapy groups (Table 3). Expression of the miRNA-15a-5p and -200a-3p were significantly decreased in all patients receiving therapy, even when investigating progressors and non-progressors separately.

### 3.5. miRNAs Indicative of Progression 

Logistic regression was used to investigate the predictive ability of miRNA expression levels on the cancer progression status. When the logistic regression model fit the data with miRNA expression only, miR-203a-3p (*p* = 0.054) and miR-205-5p (*p* = 0.080) trended towards significance but none of the miRNAs had a statistically significant effect (*p*-values < 0.050) in predicting the progression status.

Since several of the miRNA expression levels were significantly altered within treatment groups, we included two additional logistic regression models when analyzing the predictive status of the miRNAs. One model included miRNA expression levels and therapy status while the second model incorporated miRNA expression levels, therapy status and their interaction. Although miR-203a-3p (*p* = 0.054) and miR-205-5p (*p* = 0.068) trended toward significance, none of the miRNAs showed a statistically significant effect in predicting progression when miRNA expression levels were combined with treatment. The second model, which involved combining the therapy status of each group and their interaction, revealed miR-30b-5p with significant effect in predicting progression (*p* = 0.044) and miR-203a-3p (*p* = 0.054), miR-20a-5p (*p* = 0.054) and miR-205-5p (*p* = 0.065) all trending toward significance.

**Figure 2:**
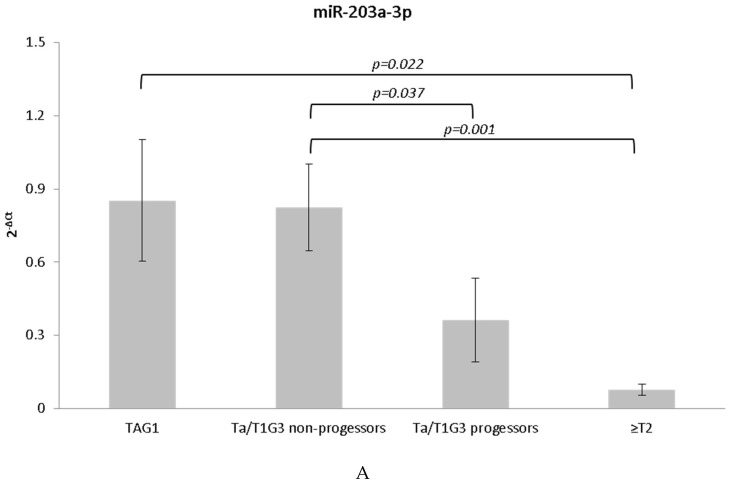

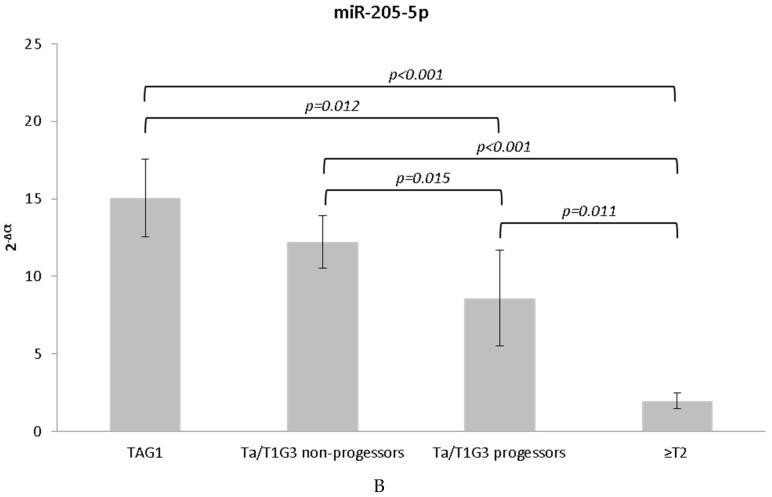
Normalized qRT-PCR expression levels. Expression correlated with the degree of invasiveness of the tumor samples for (**A**) miR-203a-3p and (**B**) miR-205-5p. Error bars represent the standard error of the mean. Significant differences are indicated with brackets; all remaining comparisons were not significant at *p* < 0.050.

As expression levels of miR-203a-3p and miR-205-5p appeared to be reduced in the group that progressed to invasive disease, we analyzed whether the expression levels of these two miRNAs were reduced in invasive tumors when compared to non-muscle invasive tumors. For this analysis we examined 26 cases representing TaG1 and 31 cases of muscle invasive (≥T2) UCB (Supplemental Materials Table S2), in addition to the expanded sample set. Results of this comparison revealed a decrease in the expression levels of miR-203a-3p and miR-205-5p correlated with the degree of invasiveness of the tumor samples (Figure 2) whereby TaG1 lesions expressed the highest levels of miR-203a-3p and miR-205-5p over the ≥T2 invasive lesions.

### 3.6. miRNAs Indicative of Mortality 

Logistic regression was used to investigate the predictive ability of all miRNA expression levels tested for cancer specific and overall mortality. When the logistic regression model fit the data with miRNA expression only, miR-412-3p was significant in predicting overall mortality (*p* = 0.020), and miR-412-3p and miR-32-5p were significant in predicting cancer specific mortality (*p* = 0.037 and *p* = 0.049 respectively).

### 3.7. Time to Progression Analyses 

The Cox proportional hazards model was used to investigate whether or not a significant change in specific miRNA expression levels corresponded with time to progress to muscle invasive disease. Multivariate Cox regression analysis of 35 miRNAs showed two miRNAs, miR-205-5p and miR-203a-3p that were significantly associated with time to progression (miR-205-5p: hazard ratio (HR), 0.789; *p* = 0.014; miR-203: HR, 0.854; *p* = 0.027). Both of these miRNAs were found to be downregulated in the progressor group and for each two-fold increase in the miRNA levels, instantaneous risk of progression decreases by 21% and 15% for miR-205-5p and miR-203a-3p, respectively. 

**Figure 3:**
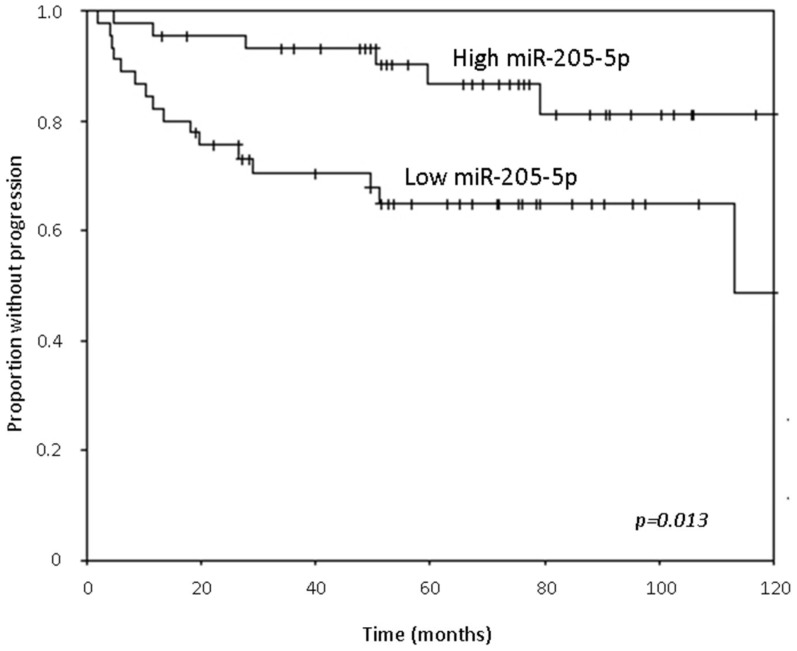
Proportion of patients that did not progress over 10 years, stratified by median miR-205-5p expression.

Kaplan-Meier analysis of time to progression revealed that higher levels of miR-205-5p were associated with a higher probability of non-progression (*p* = 0.013, Figure 3). The median time to progression in the group with low expression of miR-205-5p was 113 months compared to the high expression group where this time was not reached. In addition, higher levels of miR-17-5p and miR-20a-5p were also associated with a higher probability of non-progression (*p* = 0.048 and *p* = 0.031, respectively).

### 3.8. Time to Death Analyses

Cox proportional hazards modeling analyzing the non-progressor and progressor groups together as well as the progressor group alone revealed that no miRNAs were significant in predicting time to death from any cause (overall survival). Cox proportional hazards analysis was also used to investigate if a significant change in miRNA expression levels corresponded with the hazard of death from cancer when non-progressors and progressors were combined. Multivariate Cox regression analysis showed miR-412-3p and -224-5p were significantly associated with time to death from cancer (miR-412-3p: hazard ratio (HR), 1.45; *p* = 0.046; miR-224-5p: HR, 1.32; *p* = 0.046). For each 2-fold increase in miR-412-3p and -224-5p, the probability of survival decreases and the instantaneous risk of death from cancer increases by 45% and 32%, respectively.

## 4. Discussion

There are 40,000 new cases of NMI bladder tumors in the United States per year with >500,000 people living with this disease. The maintenance of such a patient population occurs at considerable cost to the healthcare system. Within this group of NMI tumors 45% are predicted to have a recurrence in the first twelve months and 3%–15% will likely progress to muscle invasive disease [45]. The goal of current treatment is to reduce risk of recurrence and progression with impact on survival. The treatment strategy is based on risk stratification where the number of tumors, tumor size, prior recurrence rate, stage, presence or absence of concomitant CIS and grade, all contribute to the evaluation of risk to progression. Tumors considered at high risk are usually treated with TURBT (with the option of a second resection) and intravesical therapy followed by close surveillance. Alternatively, some authors propose that high risk T1G3 patients should promptly undergo a radical cystectomy. Since the survival benefit of the latter is not yet proven, the conservative option is currently the standard of care. 

The experimental platform for this analysis emanates from the miRNA expression microarray profiling data and for this reason we selected tumor samples with Ta/G3 or T1/G3 lesions in the absence of additional clinical changes e.g., carcinoma in situ. Extension of this tumor group to further validate findings from the microarray profiles included NMI lesions with concurrent CIS and/or prior intravesical treatment. Such samples were included because of the lack of numbers of patients exhibiting non-invasive lesions alone. Such samples sully the analysis and at the same time expand consideration of the role of different clinical parameters on miRNA expression influencing the behavior of tumors emanating from the NMI group.

When using a 1.5-fold cut-off, the microarray analysis revealed 19 miRNAs were differentially expressed between the non-progressor and progressor groups, and five of these were significantly differentially expressed based on qRT-PCR results of the discovery sample set. A more extensive analysis of the expanded sample set for 35 miRNAs revealed that eighteen were significantly down-regulated in the progressor group. Six of these were also identified previously by this group as being down-regulated in muscle invasive as compared to non-muscle invasive disease [39]. Interestingly, many of these miRNAs have also been linked to the epithelial-mesenchymal transition (EMT) [40,41,42] including miR-31-5p, -200a-3p, -200b-3p, -203a-3p and -205-5p. MiR-203a-3p is implicated in keratinocyte differentiation [46] and the stem cell phenotype [47] including increased expression of ABL-1 and BCR-ABL-1 [48] whilst miR-205-5p is considered part of the miR-200 family linked to EMT [46], an event previously recorded in bladder tumorigenesis [38,39]. 

Several studies have implicated miRNAs as prognostic markers for UCB, and the study by Rosenberg et al. [37] is of particular interest to the work presented here. Their study also identified miRNAs associated with progression and survival in tumors from patients that initially presented with non-invasive disease. Several of the miRNAs they identified with reduced expression between progressors and non-progressors were also differentially expressed in this microarray analysis, including miR-29c-3p, -31-5p, -141-3p, and -205-5p. The qRT-PCR analysis of our expanded sample set similarly noted significantly decreased expression of these four miRNAs. Conversely, they found increased expression for miR-17-5p, -19b-3p, -20a-5p, and -106a-5p. However, the present data reflect a sample set restricted to only high grade samples, and included cases with CIS and/or intravesical therapy. Furthermore, some of the discrepancies between our study and the Rosenberg study may be accounted for by the differences in analytical platforms. Nonetheless, miR-205-5p stands out in both studies as a marker of progressive UCB. Some of the known targets of miR-205-5p include ZEB1/2 [40], PTEN [49], HER3 [50], SHIP2 [51], VEGFA [52] and E2F1, E2F5, and PKCε [53].

In the present study additional miRNAs have also been associated with progression as well as survival. The miRs -17-5p, -20a-5p, -30b-5p and -203a-3p were associated with progression, and miRs -19b-3p, -32-5p, -224-5p, and -412-3p were related to cancer-specific survival. Many of these have been reported to play a role in carcinogenesis. Upregulation of the miR-17-5p cluster, which includes miR-17-5p and -20a-5p, has been shown to inhibit progression of colorectal carcinoma [54]. This correlates with the higher levels of these miRNAs observed in this study for non-progressing tumors. Likewise, suppression of tumorigenesis by miR-203a-3p and -205-5p has been identified in numerous studies of cancer including UCB [55,56], and their use as clinical markers and in potential treatment strategies needs to be championed. 

Moreover, this study provided an elemental examination of miRNAs differentially expressed between tumor specimens treated with or without intravesical therapy. This is of importance because the mechanism of several miRNAs implicated in UCB has been reported, and in some cases their use as potential therapies has been proposed. Of particular note, recently Inamoto et al [57] administered miR-145-5p in an intravesical orthotopic mouse model, and observed a 76% decrease in tumor growth along with increased survival. In the present study miR-145-5p was observed to be differentially expressed in tumors of patients that received intravesical therapy, and others noted here likely warrant similar investigation as therapeutic agents. 

In this study we have shown the link of altered expression in different miRNAs indicative of disease progression in non-muscle invasive bladder tumors providing tools for the urologist on which to base clinical management decisions in this subgroup of patients. Secondarily, we have identified miRNA panels whose expression profile is affected in their therapeutic response. This provides a platform for future studies to identify definitive miRNA profiles indicative of response to treatment, and bolsters the evidence of a role for miRNA-based therapies in UCB. Further validation in a multi-institutional study is warranted.

## 5. Conclusions

We have identified miRNAs associated with a progressive phenotype and survival in UCB, and several of these miRNAs have been linked with EMT. Specifically, levels of miR-203a-3p and miR-205-5p correlated with the degree of invasiveness of the tumor samples, and both miRNAs were significantly associated with time to progression. Two miRNAs (miR-412-3p and miR-224-5p) were significantly associated with time to death from UCB. In addition, a preliminary analysis comparing patients with and without intravesical therapy revealed specific miRNAs differentially expressed between these groups. These could serve as a basis for further research into potential therapeutic agents.

## Figures and Tables

**Table 1:** Clinical profile of patients. 1A: discovery sample set used in the microarray analysis and the initial qRT-PCR validation. 1B: expanded sample set of patients used in qRT-PCR analysis. mo = months; SD = standard deviation; UW = University of Wisconsin.

**Table 1A: Discovery Sample Set**	**Non-Progressor**	**Progressor**
*n*	10	7
TaG3/T1G3 (*n*/*n*)	5/5	4/3
Male (%)	70	86
Age (mean years ± SD)	71 ± 8	73 ± 7
Time to progression (mean mo ± SD)	n/a	34 ± 37
Time to follow-up (mean mo ± SD)	88 ± 29	55 ± 43
**Table 1B: Expanded Sample Set**	**Non-Progressor**	**Progressor**
*n*	67	22
TaG3/T1G3 (*n*/*n*)	37/30	7/15
Male (%)	78	86
Age (mean yrs ± SD)	69 ± 9	73 ± 7
Time to progression (mean mo ± SD)	n/a	25 ± 26
Time to follow-up (mean mo ± SD)	73 ± 32	49 ± 33
Intravesical therapy (*n*, %)	11, 16%	5, 23%
Status (alive, %)	49, 73%	10, 45%
Site (Lahey/UW)	53/14	18/4

**Table 2:** Discovery sample set: MicroRNAs (miRNAs) differentially expressed between the non-progressor and progressor groups. Negative fold change values indicate expression was higher in non-progressors. Microarray results of the fold change in expression are based on comparing pooled samples from each group. The qRT-PCR results (fold change, means, std. dev., and *p*-value) reflect an analysis of individual samples for each group (non-progresor group *n* = 10 and progressor group *n* = 7). Mean values are expressed as 2^−∆Ct^. std. dev. = standard deviation.

microRNA	Microarray Fold Change	qRT-PCR					
		Fold Change	*p*-Value	Non-Progressors		Progressors	
				Mean	Std. Dev.	Mean	Std. Dev.
hsa-miR-125b-5p	2.46	3.28	0.057	0.64	1.10	2.09	1.54
hsa-miR-145-5p	2.09	3.51	0.031	0.10	0.10	0.37	0.25
hsa-miR-223-3p	1.82	4.32	0.102	0.26	0.23	1.11	1.16
hsa-miR-143-3p	1.70	1.96	0.103	0.15	0.20	0.30	0.15
hsa-miR-338-5p	1.69	2.96	0.274	0.00	0.00	0.01	0.02
hsa-miR-31-5p	−2.71	−2.24	0.143	5.24	5.29	2.34	2.10
hsa-miR-203a-3p	−2.59	−4.75	0.025	0.54	0.49	0.11	0.13
hsa-miR-141-3p	−2.44	−3.04	0.008	2.18	1.25	0.72	0.71
hsa-miR-200a-3p	−2.25	−2.19	0.021	5.12	2.85	2.34	1.56
hsa-miR-205-5p	−2.12	−3.05	0.034	15.97	12.90	5.24	5.06
hsa-miR-429	−2.09	−1.55	0.366	0.63	0.62	0.41	0.36
hsa-miR-29c-3p	−1.92	−1.61	0.339	1.02	1.08	0.63	0.50
hsa-miR-20a-5p	−1.88	−1.43	0.299	1.79	1.15	1.25	0.91
hsa-miR-29b-3p	−1.86	−1.87	0.064	0.36	0.23	0.19	0.10
hsa-miR-19b-3p	−1.77	−2.13	0.080	1.73	1.36	0.81	0.59
hsa-miR-17-5p	−1.72	−1.29	0.443	1.87	1.12	1.45	1.04
hsa-miR-106a-5p	−1.71	−1.29	0.446	1.67	0.99	1.30	0.97
hsa-miR-200b-3p	−1.63	−1.34	0.335	4.98	2.29	3.70	2.76
hsa-miR-30e-5p	−1.58	−1.23	0.547	0.88	0.77	0.72	0.29

**Table 3:** Relative expression of miRNAs differentially expressed between patients of the expanded sample set with or without intravesical therapy. Negative fold change values indicate expression was higher in patients without therapy. Mean values are expressed as 2^−∆Ct^. std. dev. = standard deviation.

**All Patients of the Expanded Sample Set (*n* = 88)**			**Without Therapy**		**With Therapy**	
miRNA	fold change	*p*-value	mean	std. dev.	mean	std. dev.
hsa-miR-15a-5p	−3.00	<0.001	0.37	0.32	0.12	0.05
hsa-miR-20a-5p	−2.71	<0.001	1.40	1.38	0.52	0.29
hsa-miR-21-5p	−2.24	0.003	8.83	10.31	3.93	3.86
hsa-miR-200a-3p	−3.58	<0.001	3.65	5.25	1.02	1.06
hsa-miR-1308	−2.79	<0.001	171.92	191.95	61.63	42.86
**Within Progressors Only (*n* = 21)**			**Without Therapy**		**With Therapy**	
miRNA	fold change	*p*-value	mean	std. dev.	mean	std. dev.
hsa-miR-15a-5p	−2.32	0.004	0.33	0.15	0.14	0.06
hsa-miR-32-5p	−3.13	0.010	0.01	0.01	0.00	0.00
hsa-miR-141-3p	−3.02	0.011	0.88	0.77	0.29	0.19
hsa-miR-200a-3p	−2.31	0.011	2.11	1.37	0.91	0.48
hsa-miR-200c-3p	−2.05	0.014	10.51	6.26	5.12	2.33
hsa-miR-429	−2.17	0.034	0.32	0.28	0.15	0.07
**Within Non-Progressors Only (*n* = 67)**			**Without Therapy**		**With Therapy**	
miRNA	fold change	*p*-value	mean	std. dev.	mean	std. dev.
hsa-miR-15a-5p	−3.33	<0.001	0.38	0.36	0.11	0.05
hsa-miR-17-5p	−2.56	<0.001	1.82	1.62	0.71	0.46
hsa-miR-19a-3p	−2.93	<0.001	0.24	0.26	0.08	0.06
hsa-miR-19b-3p	−2.20	0.004	1.58	1.73	0.72	0.57
hsa-miR-20a-5p	−2.99	<0.001	1.55	1.48	0.52	0.33
hsa-miR-21-5p	−3.21	<0.001	9.87	11.36	3.08	3.52
hsa-miR-29b-3p	−2.37	0.008	0.42	0.63	0.18	0.09
hsa-miR-30b-5p	−1.64	0.012	3.10	3.07	1.89	0.75
hsa-miR-30e-5p	−1.90	0.005	1.36	1.31	0.71	0.28
hsa-miR-32-5p	−2.23	0.018	0.01	0.02	0.01	0.00
hsa-miR-106a-5p	−2.27	0.002	1.66	1.51	0.73	0.52
hsa-miR-135a-5p	−6.99	0.003	0.08	0.14	0.01	0.01
hsa-miR-141-3p	−2.42	0.005	1.41	1.48	0.59	0.64
hsa-miR-200a-3p	−3.90	<0.001	4.11	5.88	1.06	1.23
hsa-miR-200b-3p	−2.27	0.003	4.68	4.24	2.07	1.95
hsa-miR-203a-3p	−2.99	0.010	0.93	1.58	0.31	0.32
hsa-miR-301a-3p	−5.56	0.012	0.09	0.20	0.02	0.01
hsa-miR-429	−3.72	0.002	0.56	0.86	0.15	0.18
hsa-miR-1308	−2.81	0.001	177.74	208.59	63.22	37.36

## Supplementary Materials

The following are available online at www.mdpi.com/2073-4425/8/2/77/s1, Table S1: Relative expression within the expanded sample set for the 35 miRNA tested by qRT-PCR, Table S2: Clinical profile of the additional patients analyzed for miR-203-3p and miR-205-5p and compared to the non-progressors and progressors.
