# Supplementary material for: MicroRNA Expression Profile Identifies High Grade, Non-Muscle-Invasive Bladder Tumors at Elevated Risk to Progress to an Invasive Phenotype
Source: Genes (Basel). 2017 Feb 17;8(2):77. doi: 10.3390/genes8020077 (PMC5333066; doi:10.3390/genes8020077)
Supplement: Supplementary file 1 [file genes-08-00077-s001.docx]

Supplementary Materials: MicroRNA Expression Profile Identifies High Grade, Non-Muscle-Invasive Bladder Tumors at Elevated Risk to Progress to an Invasive Phenotype

Sara M. Lenherr, Sheaumei Tsai, Brasil Silva Neto, Travis B. Sullivan, Cara B. Cimmino,
Tanya Logvinenko, Jason Gee, Wei Huang, John A. Libertino, Ian C. Summerhayes and Kimberly M. Rieger-Christ

**Table S1.** Relative expression within the expanded sample set for the 35 miRNA tested by qRT-PCR. Negative fold change values indicate expression was higher in non-progressors. Mean values are expressed as 2^-∆Ct^, std. dev.: standard deviation.

| **microRNA** | **Fold Change** | ***p*-Value** | **Non-Progressors** | | **Progressors** | |
| --- | --- | --- | --- | --- | --- | --- |
|  |  |  | **Mean** | **Std. dev.** | **Mean** | **Std. dev.** |
| hsa-miR-15a-5p | −1.17 | 0.419 | 0.33 | 0.34 | 0.29 | 0.16 |
| hsa-miR-17-5p | −1.58 | 0.039 | 1.63 | 1.55 | 1.03 | 0.78 |
| hsa-miR-19a-3p | −2.07 | 0.001 | 0.22 | 0.25 | 0.11 | 0.06 |
| hsa-miR-19b-3p | −1.92 | 0.002 | 1.44 | 1.63 | 0.75 | 0.43 |
| hsa-miR-20a-5p | −1.73 | 0.028 | 1.38 | 1.41 | 0.80 | 0.70 |
| hsa-miR-21-5p | −1.57 | 0.050 | 8.75 | 10.76 | 5.57 | 4.21 |
| hsa-miR-29b-3p | −1.80 | 0.030 | 0.38 | 0.58 | 0.21 | 0.14 |
| hsa-miR-29c-3p | −1.79 | 0.029 | 1.54 | 2.21 | 0.86 | 0.65 |
| hsa-miR-30b-5p | −1.66 | 0.004 | 2.90 | 2.86 | 1.75 | 0.79 |
| hsa-miR-30e-5p | −1.57 | 0.017 | 1.25 | 1.22 | 0.79 | 0.31 |
| hsa-miR-31-3p | −1.95 | 0.038 | 0.26 | 0.38 | 0.13 | 0.12 |
| hsa-miR-31-5p | −1.40 | 0.242 | 2.56 | 3.94 | 1.83 | 1.80 |
| hsa-miR-32-5p | −2.02 | 0.025 | 0.01 | 0.02 | 0.01 | 0.01 |
| hsa-miR-99a-5p | −1.09 | 0.705 | 1.01 | 1.13 | 0.92 | 0.79 |
| hsa-miR-100-5p | −1.01 | 0.956 | 0.89 | 0.89 | 0.88 | 0.77 |
| hsa-miR-106a-5p | −1.63 | 0.029 | 1.51 | 1.43 | 0.92 | 0.71 |
| hsa-miR-125b-5p | −1.27 | 0.245 | 3.69 | 3.57 | 2.91 | 2.30 |
| hsa-miR-135a-5p | −3.80 | 0.010 | 0.07 | 0.13 | 0.02 | 0.03 |
| hsa-miR-141-3p | −1.67 | 0.033 | 1.28 | 1.41 | 0.77 | 0.73 |
| hsa-miR-143-3p | −1.24 | 0.314 | 0.35 | 0.34 | 0.28 | 0.23 |
| hsa-miR-144-3p | −2.80 | 0.203 | 0.02 | 0.08 | 0.01 | 0.01 |
| hsa-miR-145-5p | −1.35 | 0.218 | 0.71 | 0.70 | 0.53 | 0.55 |
| hsa-miR-200a-3p | −1.92 | 0.020 | 3.61 | 5.51 | 1.88 | 1.33 |
| hsa-miR-200b-3p | −1.48 | 0.038 | 4.25 | 4.07 | 2.87 | 1.94 |
| hsa-miR-200c-3p | −1.44 | 0.041 | 13.67 | 12.36 | 9.48 | 6.07 |
| hsa-miR-203a-3p | −3.32 | 0.007 | 0.82 | 1.47 | 0.25 | 0.49 |
| hsa-miR-205-5p | −2.01 | 0.003 | 12.18 | 13.98 | 6.06 | 5.03 |
| hsa-miR-223-3p | −1.05 | 0.860 | 0.93 | 1.30 | 0.88 | 0.84 |
| hsa-miR-224-5p | −1.22 | 0.617 | 0.21 | 0.31 | 0.17 | 0.25 |
| hsa-miR-301a-3p | −2.01 | 0.150 | 0.08 | 0.18 | 0.04 | 0.05 |
| hsa-miR-338-5p | −1.00 | 0.995 | 0.01 | 0.01 | 0.01 | 0.01 |
| hsa-miR-412-3p | 1.15 | 0.705 | 0.04 | 0.06 | 0.04 | 0.05 |
| hsa-miR-429 | −1.72 | 0.072 | 0.50 | 0.81 | 0.29 | 0.26 |
| hsa-miR-451a | −5.57 | 0.221 | 5.17 | 24.76 | 0.93 | 1.60 |
| hsa-miR-1308 | −1.22 | 0.476 | 158.29 | 195.14 | 130.08 | 118.25 |

**Table S2.** Clinical profile of the additional patients analyzed for miR-203-3p and miR-205-5p and compared to the non-progressors and progressors.

| **Additional Samples** | **TaG1** | **Invasive UCB** |
| --- | --- | --- |
| n | 25 | 31 |
| Male (%) | 73 | 61 |
| Age (mean years ± SD) | 67 ± 15 | 66 ± 10 |
| Time to follow-up (mean mo ± SD) | 71 ± 39 | 28 ± 42 |

Mo = months; SD = standard deviation; These patients were adapted from Wszoleck et.al. 2011.

**References**

1. Wszoleck, M.; Rieger-Christ, K.M.; Kenney, P.A.; Gould, J.J.; Silva Neto, B.; LaVoie, A.K.; Logvinenko, T.; Libertino, J.A.; Summerhayes, I.C. MicroRNA expression profiles and biomarkers linked to the invasive bladder tumor phenotype. *Urol. Oncol.* **2011**, *29*, 794–801.
